# Numerical Simulation of the Rheological Behavior of Nanoparticulate Suspensions

**DOI:** 10.3390/ma13194288

**Published:** 2020-09-25

**Authors:** Benedikt Finke, Arno Kwade, Carsten Schilde

**Affiliations:** Institute for Particle Technology, Technische Universität Braunschweig, Volkmaroder Str. 5, 38104 Braunschweig, Germany; a.kwade@tu-bs.de (A.K.); c.schilde@tu-bs.de (C.S.)

**Keywords:** nano-composites, CFD-DEM coupling, viscosity simulation, rheology, suspension viscosity, surface forces, viscosity modelling, polymer viscosity, epoxy resin, nanoparticles, composite materials

## Abstract

Nanoparticles significantly alter the rheological properties of a polymer or monomeric resin with major effect on the further processing of the materials. In this matter, especially the influence of particle material and disperse properties on the viscosity is not yet understood fully, but can only be modelled to some extent empirically after extensive experimental effort. In this paper, a numerical study on an uncured monomeric epoxy resin, which is filled with boehmite nanoparticles, is presented to elucidate the working principles, which govern the rheological behavior of nanoparticulate suspensions and to simulate the suspension viscosity based on assessable material and system properties. To account for the effect of particle surface forces and hydrodynamic interactions on the rheological behavior, a resolved CFD is coupled with DEM. It can be shown that the particle interactions caused by surface forces induce velocity differences between the particles and their surrounding fluid, which result in increased drag forces and cause the additional energy dissipation during shearing. The paper points out the limits of the used simulation method and presents a correction technique with respect to the Péclet number, which broadens the range of applicability. Valuable information is gained for a future mechanistic modelling of nanoparticulate suspension viscosity by elucidating the interdependency between surface forces, shear rate and resulting drag forces on the particles.

## 1. Introduction

Numerous reports exist on the strong effect of nanoparticles on the rheological behavior of nanoparticulate suspensions [1]. Surface forces are considered to be the dominating cause of the strong observable increases in viscosity [2,3]. In the case of polymer suspensions, an immobilization of molecules is considered to influence the viscosity as well [4]. Despite extensive and persistent effort of the scientific community to develop methods and models to predict the rheological properties of suspensions [5], the range of applicability of such methods and models is still very limited [6,7]. This particular work is done in the focus of the production of lightweight fiber reinforced nano-composite structures for aerospace applications. Here, boehmite nanoparticles are added to an uncured monomeric epoxy resin in order to improve the mechanical properties [8] of the matrix material since they often limit the mechanical performance of fiber reinforced composites. The key obstacle in this field is the fact that the beneficial effect on the mechanical properties comes with the disadvantage of a steep increase in viscosity, which has a strong effect on the processing [8,9] of the material and impedes the injection of the uncured epoxy resin into the fiber layers during impregnation. Studies on the material system, which is regarded in this study, show that difficulties during impregnation arise from shortened flow-length of the material in the fiber layers. They originate from an increased pressure drop along the fluid path through the layers, which is caused by the increased viscosity [10]. In addition, filtering effects can amplify this effect, especially when coarse particles block the flow or inter-fiber spacing is small [11]. Tailoring the particulate system for a maximum gain in mechanical strength and minimized viscosity increase during production requires insight on the underlying working principles causing the viscosity increase. To this day, most models available for the characterization of suspension viscosities are empirical or semi-empirical and require extensive experimental effort, as model parameters originate from the approximation of measured experimental data. This is especially true for models dealing with nanoparticulate systems [7]. Furthermore, most analytical models do not cover any disperse properties other than solids content and disregard the effect of material properties, even though their significance has been proven numerous times [1]. This is why with the development of capable numerical methods for the investigation of such physical phenomena, numerical simulations have been employed to predict the viscosity of particle-laden fluids.

This work applies coupled CFD-DEM simulations to gain insight on the working principles causing the rheological behavior of nanoparticulate suspensions. Thus, the present work seeks to open up the prospect of being able to mechanistically model the rheological properties of nanoparticulate suspensions in a broader range than possible today.

## 2. State of the Art

Independent from the specific method, numerical simulation of rheological properties focusses on the combined depiction of solid-fluid interactions (hydrodynamic interactions) and the interaction between particles. The first methods to be employed for this purpose were Stokesian Dynamics [12] and Molecular Dynamics (MD) simulation of multiple phases. In 1985, Brady and Bossis [13] proved the capability of Stokesian Dynamics to model non-Newtonian behavior of fluids, taking advantage of the fact that in simulation the individual sources of interaction can be studied independent from another. Doi and Chen [14] employed a Stokesian Dynamics simulation set up to study changes in the particle-cluster structure with respect to the applied shear rate in order to elucidate the working principles causing shear thinning behavior of colloidal suspensions. Due to the insight gained on the colloidal structure of the particles in the shear flow, they were able to suggest that the rheological behavior of the suspension is not governed by the size or shape of the dynamically forming and disintegrating structure of particle clusters, but rather on the size of sub-structures comprised of a few particles with stronger binding. Stokesian Dynamics were also proven to be applicable [15] for obtaining otherwise non-accessible parameters required for an analytical viscosity model and were used to verify model assumptions and confirm experimentally obtained parameters. This enabled the successful modelling of nanoparticle suspensions over a broad range of solids contents. Yamamoto et al. [16] extended the method to account for the dynamics of rod-like particles and proved its capability to account for the additional energy dissipation due to the non-spherical shape of the particles.

Woodcock [17] applied a coarse-grained non-equilibrium Molecular Dynamics simulation to model the shear thinning behavior of 1 μm sized PVC particles at high solids contents ($c_{v}=0.55$) and received good agreement with experimental findings. With a less coarse-grained approach, Starr et al. [18] were able to depict that shear thinning behavior depends on the dispersion state of the suspension. They also confirmed the results of Doi and Chen [14] concerning the subordinated effect of the size and shape of weak clusters on the rheological behavior and pointed out that the interaction between particle surface and fluid molecules [19] influences the structure of such molecules, which might result in an altered rheological behavior of the suspension.

MD simulations excel at elucidating effects on molecular level, but are quite costly. Disspative Particle Dynamics (DPD) represent fluid elements with discrete spheres, which mimic the behavior of a fluid. This reduces the computational effort significantly. With this method, Koelman et al. [20] were able to depict the relative viscosity of a suspension of spheres in the sub-micron size range, based on hydrodynamic and Brownian fluctuating forces. Boek et al. [21] investigated the effect of particle size and the relevance of individual mechanisms of interaction and compared it to existing models.

A further step in abstracting the fluid phase of a suspension is taken, when exclusively employing the Discrete Element Method (DEM) to investigate the rheology of suspensions. In recent years, this method proved to be very capable of illuminating rheological phenomena in suspensions with a high solids content of micron scale particles [22,23]. Ness et al. [24] employed DEM simulation in which contact models mimicked hydrodynamic lubrication forces, frictional and contact forces as well as a repulsive particle interaction, which they simplified to a linear spring model. They could show that this method allows depicting the flow regime transition in high solids content suspensions ($c_{v}>0.45$). This method even allowed the derivation of a constitutive model with respect to the dominating sources of interaction, which were found to be characteristic for the respective regimes. The method also provided rheological regime maps for these suspensions, which are otherwise hard to model [25]. Lin et al. [26] employed a similar set up, which differed mainly by a fully resolved repulsive electrostatic force between the particles. They could show that the rheological phenomenon of shear thickening can be attributed to contact forces rather than to the formation of hydrodynamic clusters by quantifying the contribution of the individual types of interaction to the overall viscosity during shear reversal experiments. Pähtz et al. [27] regarded lubrication forces and a viscous drag force in a DEM simulation. The viscous drag force was calculated as done in Stokesian Dynamics simulations. They showed that the flow curves of many suspensions and flow conditions could be characterized by a friction coefficient, which scales with the Péclet number and the contact models friction parameter.

It is noticeable that DEM can only be used alone, if the suspensions have a high solids content of coarse particles with pronounced inertia, since friction-, contact-, and lubrication forces were found to dominate in this region. In contrast, nanoparticulate suspensions already exhibit strong viscosity increases at low solids contents. Consequently, friction- and contact forces cannot be expected to have a strong effect. Instead, inter-particle surface forces and particle-fluid interactions must be evaluated with great care.

Computational Fluid Dynamics (CFD) is a very common method to solve fluid flow. A coupling of CFD with DEM can therefore yield the required information on the interaction of fluid and solid phase. Yoshida et al. [28] studied the viscosity of silica particles dispersed in a mixture of ethylene glycole and water. The particle size was varied from micron to nano scale. Simulations were conducted by coupling CFD and DEM on the level of Direct Numerical Simulation (DNS). For DNS, the chosen mesh size resolves the fluid flow down to the level of the Kolmogorov length scale, i.e., to the size of the smallest eddy in a turbulent flow and hence yields an exact solution of such flow. The method yielded good agreement between simulation and experiment with respect to particle size and solids content. All DLVO forces were regarded as contact models in the DEM. Smuts [29] chose an unresolved way to couple CFD and DEM to simulate the viscosity of suspensions from water and micron particles. In unresolved CFD-DEM simulations, drag models are employed to account for the drag forces acting on the particles. In this case, the CFD cells can be larger than the particles, allowing the depiction of larger systems. The flow field around the particles however remains unresolved.

In a rotatory viscometer, the shear viscosity of a fluid or a suspension is measured by shearing a fluid sample in a defined gap and measuring the shear stress acting on the wall of the measurement cell by monitoring the torque acting on the rotating geometry. Most of the simulations mentioned above were performed with periodic boundary conditions, which prevents any estimation of a shear stress on a wall, since no walls exist. Independent from the specific simulational method, in most studies the viscosity of the suspension is estimated by quantifying the stress acting in the system. The procedure was developed by Batchelor [30], who modified a method of averaging properties in the volume of a disperse systems [31]. The shear stress $\tau_{xy}$ in the x-y plane perpendicular to the velocity gradient in z-direction is taken to be equal to the difference between the mean stress tensor ${\bar{\sigma }}_{xy}$ and the tensor of momentum flux ${\bar{m}}_{xy}$ as given in Equation (1).

(1) $$\tau_{xy}={\bar{\sigma }}_{xy}-{\bar{m}}_{xy}$$

In a stationary system, momentum flux balances to zero, which is why the expression can be simplified to Equation (2a). The method assumes the average value of a parameter to be equal to the sum of all local values in a volume *V*, divided by the volume as presented in Equation (2b).

(2a) $$\begin{array}{cc} \tau_{xy} & ={\bar{\sigma }}_{xy} \end{array}$$

(2b) $$\begin{array}{cc} \; & =\frac{1}{V}\cdot \sum_{i}\sigma_{i} \end{array}$$

(2c) $$\begin{array}{cc} \; & =\frac{1}{V}\cdot \left(\sum_{i\in fl}\sigma_{i}+\sum_{i\in particle}\sigma_{i}\right) \end{array}$$

(2d) $$\begin{array}{cc} \; & =\frac{1}{V}\cdot \left(\sum_{i\in fl}\sigma_{Fl,i}+\sum_{particle}\sigma_{particle}\right) \end{array}$$

This procedure allows the individual assessment of each phase in the system Equation (2c), whereby in the last instance a summation of the shear stress contributions of all particles in the system can take place (compare Equation (2d)). The aforementioned stress contributions are referred to as ‘stresslets’.

Smuts [29] chose a different approach and conducted his simulations with a set up in which the boundaries parallel to the shear flow were walls. This enabled him to estimate the stress acting on these walls by first summing the contact forces $F_{wall}$ transferred to the wall by the suspended particles and then relate the value to the walls surface area $A_{wall}$ of the wall (compare Equation (3)). Additionally, the to the fluids contribution to the wall stress was regarded.

(3) $$\tau_{particles,x}=\frac{\sum F_{wall,x}}{A_{wall}}$$

## 3. Materials and Methods

**Figure 1:**
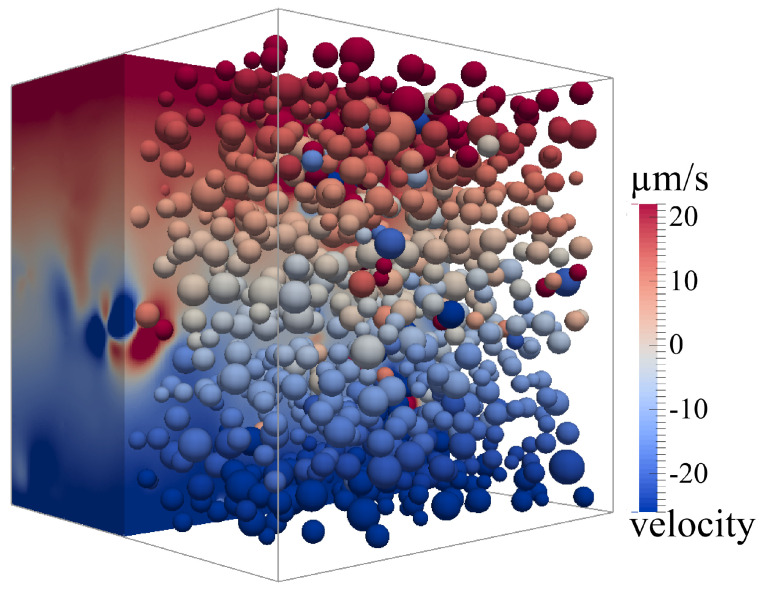
Representative volume element used in the CFD-DEM simulations.

For this numerical study, coupled CFD-DEM simulations were conducted. The simulation domain consisted of a cubic representative volume element. In a preliminary convergence study, a side length of 1.44 μm was found to be suitable to produce reproducible, system-size-independent results with little deviation between runs with varying seeds. The number of particles in the system proved to be the limiting parameter in this matter, as a certain number of particles is needed to depict a representative disperse system. Details on the convergence study are given in the Appendix A. For the CFD simulation the boundary condition of the upper and lower wall enforced a constant shear velocity with respect to the desired shear rate, as both walls were set with fixed velocities in opposite direction (compare Figure 1). All other walls were equipped with periodic boundary conditions. The particles were accounted for by the introduction of polydisperse DEM-particles in accordance with the respective solids content in each simulated case. The particle size distribution was taken from experiments via Dynamic Light Scattering (see Nolte et al. [32]). The measured log-normal distribution with more than 50 particle size intervals was reduced to 12–18 intervals. An algorithm chose the number of intervals, by reducing the resolution of the distribution while trading of the correct overall particle volume in the system and the Euclidian distance between the original and the simplified distribution.

The coupling of CFD and DEM was performed with the immersed boundary method [33], which allows the depiction of the flow field surrounding the particles. For a realistic result the particle surface must be resolved by at least eight CFD cells [34]. The simulations were performed with the academic branch of the coupling code CFDEM, which couples the DEM code LIGGGHTS with OpenFOAM. The coupling code CFDEM [34] allows coupling between fluid and particle phase in both directions, as well as particle-particle interactions. Only rotatory velocities of the particle surface are not transferred to the fluid phase and tangential flow of fluid does not change the rotatory movement of the particle.

**Figure 2:**
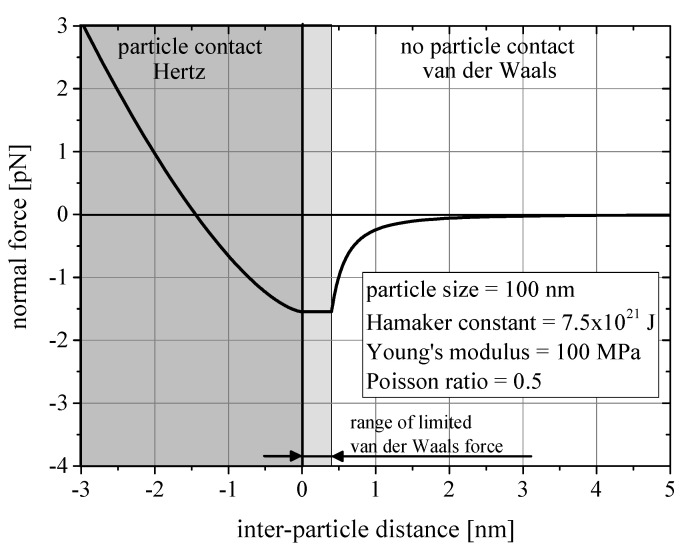
Force distance curve of the contact model for the combined representation of van der Waals forces and Hertzian contacts.

To account for the inter-particle interactions a Hertzian contact model was modified to include van der Waals type interactions before overlap of the particles. Figure 2 presents the force distance curve of the contact model for the collision of two particles of 100 nm diameter. For reasons of representation, the Young’s modulus was reduced to 100 MPa, which corresponds to a value at least two orders of magnitude below the Young’s modulus of boehmite [35]. As visible from Figure 2, at a distance of 0.4 nm, van der Waals forces where limited to a constant value to ensure the stability of the simulation, since attractive forces would tend to infinity at close particle proximity, before repulsive forces of the Hertzian contact model would be effective. The value of 0.4 nm was chosen, because it is often considered to be the minimum roughness, which even completely smooth particles exhibit and prevents particles from approaching closer. MD-based studies [36,37] on the maximum non-contact forces between silica nanoparticles when regarding Lennard-Jones potential and Born-repulsion, agreed with this value. The van der Waals forces $F_{vdW}$ where calculated with respect to the first derivative of the van der Waals potential $W_{vdW}$ (compare Equations (4) and (5)) [38]. The particle radii are denoted by *r* and the distance between the surfaces is denoted by *b*.

(4) $$\begin{array}{c} W_{vdW}=-\frac{H}{6}\left[\frac{2r_{i}r_{j}}{(2r_{i}+2r_{j}+b)b}+\frac{2r_{i}r_{j}}{\left(2r_{i}+b\right)\left(2r_{j}+b\right)}+ln\frac{(2r_{i}+2r_{j}+b)b}{\left(2r_{i}+b\right)\left(2r_{j}+b\right)}\right] \end{array}$$

(5) $$F_{vdW}=\frac{32}{3}\cdot \frac{Hr_{i}^{3}r_{j}^{3}\left(r_{i}+r_{j}+b\right)}{b^{2}{\left(2r_{i}+2r_{j}+b\right)}^{2}{\left(b^{2}+2br_{i}+2br_{j}+4r_{i}r_{j}\right)}^{2}}$$

The effect of the interstitial fluid phase on the particle-particle interaction is regarded by applying Equation (6) for the Hamaker constant *H*.

(6) $$H=\frac{3}{4}k_{B}T{\left(\frac{\epsilon_{1}-\epsilon_{3}}{\epsilon_{1}+\epsilon_{3}}\right)}^{2}+\frac{3h\nu_{e}}{16\sqrt{2}}\frac{{\left(n_{1}^{2}-n_{3}^{2}\right)}^{2}}{{\left(n_{1}^{2}+n_{3}^{2}\right)}^{3/2}}$$

The calculation of the Hamaker constant for the respective materials requires numerous material parameters, which were either measured or taken from literature. A detailed list of the input parameters and their origin is stated in Table 1. The static dielectric constant ϵ was calculated from the refractive index as given by Equation (7) [38].

(7) $$\epsilon =1+\left(n^{2}-1\right)/\left(1+\frac{\nu^{2}}{\nu_{e}^{2}}\right)$$

With the given material parameters and the use of Equation (6) a Hamaker constant of $H=7.5\times 10^{-21}\;J$ was calculated and used for the simulations.

Due to the unpolar nature of the epoxy resin, no ion double-layer can form around the particle. Consequently, electrostatic repulsive forces can be neglected. Due to the lack of repulsive forces, the particle system must be considered intrinsically instable, as no repulsive force prevents the particles from agglomerating (compare Figure 2). If the fluid phase of the system was a polar liquid like water, such forces should be regarded. The unhindered attraction of the particles leads to an artificial acceleration of the particles, which falsifies the simulations. Therefore, particle insertion was done in a way that prevented the insertion of particles in a proximity closer than 0.4 nm, which suppressed such effects. This might be considered an unjustified tempering with the structure of the system. However, during the production and preparation of the samples for the experiments, particles in immediate proximity agglomerated unless shear forces separated them. A depletion of particles in the immediate vicinity of the particle surface is therefore more likely than a direct neighbor.

The viscosity was evaluated based on the common assumption that multiple effects on the viscosity add up to the observable suspension viscosity $\eta_{susp}$. The contribution of the fluid viscosity $\eta_{fl}$, and the particle contribution $\eta_{particle}$ is regarded as stated in Equation (8).

(8) $$\eta_{susp}=\left(1-c_{v}\right)\cdot \eta_{fl}+\eta_{particle}$$

The contribution of the fluid viscosity is weighted with the fluid volume content $\left(1-c_{v}\right)$, since energy dissipation due to the inner friction of the fluid cannot take place in the part of the suspension, which is occupied by particles. The particle contribution is estimated based on the method of Batchelor [30], presented in Section 2 (see Equation (2)). The relationship between the viscosity, shear rate $\dot{\gamma }$ and shear stress is given in Equation (9).

(9) $$\eta_{particle}=\frac{\tau_{particle}}{\dot{\gamma }}=\frac{{\bar{\sigma }}_{xy}}{\dot{\gamma }}$$

The stress tensor ${\bar{\sigma }}_{xy}$ is calculated based on Equation (10), accounting for contribution from drag forces, van der Waals forces and contact forces.

(10) $${\bar{\sigma }}_{xy}=\sigma_{drag}+\sigma_{vdW}+\sigma_{contact}$$

The respective stresslet were calculated as presented in Equations (11)–(13). The particle radius is represented by $r_{i}$ and the distance between the centers of two interacting particles is given by $a_{i,j}$.

(11) $$\sigma_{drag}=\frac{1}{V}\cdot \left[\sum_{i}\left(r_{i}\cdot F_{drag,i,x}+r_{i}\cdot F_{drag,i,y}\right)\right]$$

(12) $$\sigma_{vdW}=\frac{1}{V}\cdot \left[\sum_{i}\sum_{j\neq i}\left(a_{i,j}\cdot F_{vdW,i,x}+a_{i,j}\cdot F_{vdW,i,y}\right)\right]$$

(13) $$\sigma_{contact}=\frac{1}{V}\cdot \left[\sum_{i}\sum_{j\neq i}\left(a_{i,j}\cdot F_{contact,i,x}+a_{i,j}\cdot F_{contact,i,y}\right)\right]$$

The method of Batchelor [30] can be applied, when the regarded volume can be considered representative to the system (see convergence study in Appendix A) and no significant changes to the system happen during the time of evaluation. Consequently, a steady state must be ensured before performing the stresslet evaluation. Preliminary simulation studies revealed that to reach a steady state not the simulated time was significant, but the number of coupling intervals between CFD and DEM. Consequently, the coupling interval was set to 1 and the timestep width to a very small value of $1\times 10^{-8}\;\mu$s. This limits the distance particles can travel and thus, prevents artificial agglomeration events to occur. The simulation gains the characteristics of a steady state simulation, as only slight movement allows a relaxation of the system. Steady state was reached after 900–1200 timesteps. An average from the last 125 timesteps was evaluated.

For validation of the simulation results, an epoxy-boehmite nano-composite material was chosen. The suspension consisted of uncured Araldyte LY 556 epoxy resin (HUNTSMAN) and boehmite nanoparticles HP14 (SASOL), dispersed to a median particle size $x_{50,3}$ of 75 nm. A detailed depiction of the manufacturing process can be found in Jux et al. [8].

The rheological characterization of the sample material was conducted in a rheometer (Kinexus, Malvern) with a plate-plate geometry. Rheological measurements were performed in a temperature range of 30–80 ${}^{{}^{\circ}}$C for suspension samples with solids contents between 0 and 20 wt.%. This corresponds to a volumetric solids content of 0 to approximately 10 vol.%. The CFD was equipped with the neat resin viscosity at the respective temperature. The fluid shows Newtonian behavior and a strong temperature dependency [41]. Non-Newtonian behavior of the fluid phase could be regarded by using the viscosity value at a specific shear rate as the fluid viscosity in the CFD. If local changes in the shear rate (e.g., due the presence of particles) shall be regarded, a non-Newtonian viscosity can be defined in the CFD simulation. The CFD code allows a power-law model, which calculates the viscosity based on the local velocity gradient between neighboring cells. This way also the typical rheological behavior of polymers can be regarded.

## 4. Results and Discussion

### 4.1. Applicability and Limits of the Chosen Method

**Figure 3:**
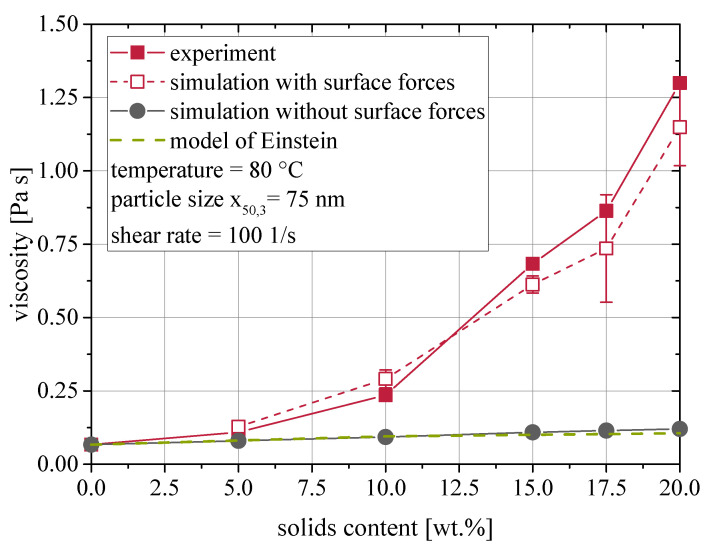
Comparison of experimental and simulated viscosities at varied solids contents.

To test the applicability of the simulation set up for the simulation of the suspension viscosities, the solids content in the suspension was varied and compared to experimental values. The results are given in Figure 3 for suspensions characterized at 80 ${}^{{}^{\circ}}$C with a shear rate of $\dot{\gamma }=100$ 1/s. Good agreement between experimental and simulated results is visible, when surface forces are regarded in the simulation, especially at low solids contents. If no van der Waals forces are regarded, the viscosity is much lower and is just slightly larger than the Einstein model [42,43] predicts. This proves the significance of surface forces for the rheological behavior of nanoparticulate suspensions. Even without surface forces, the simulated viscosity is slightly larger than Einstein’s prediction, because the dilute limit of 2 vol.% [44] is exceeded and hydrodynamic interactions lead to additional energy dissipation.

The error bars of the simulational results with surface forces indicate increasing scattering with increasing solids content. In addition, slight deviations between experimental and simulational values arise at high solids contents, as the simulation underestimates the viscosity of such suspensions. This phenomenon will be addressed later.

**Figure 4:**
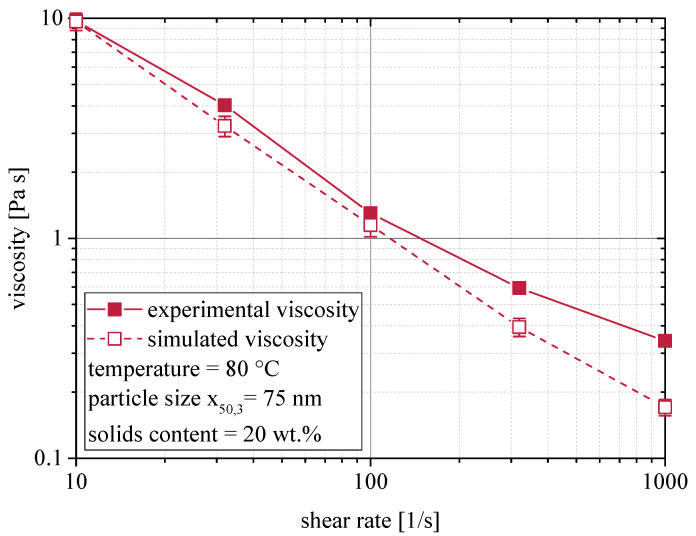
Shear rate dependency of the rheological behavior of nanoparticulate suspensions. Comparison of experimental and simulated viscosities at 80 ${}^{{}^{\circ}}$C.

In Figure 4 the results of a shear rate variation at 80 ${}^{{}^{\circ}}$C are compared for a suspension with a solids content of 20 wt.%. Again, good agreement is observed over the depicted range of shear rates. However, with increasing shear rate, simulation results tend to range below the experimental values, which causes increasing deviation.

**Figure 5:**
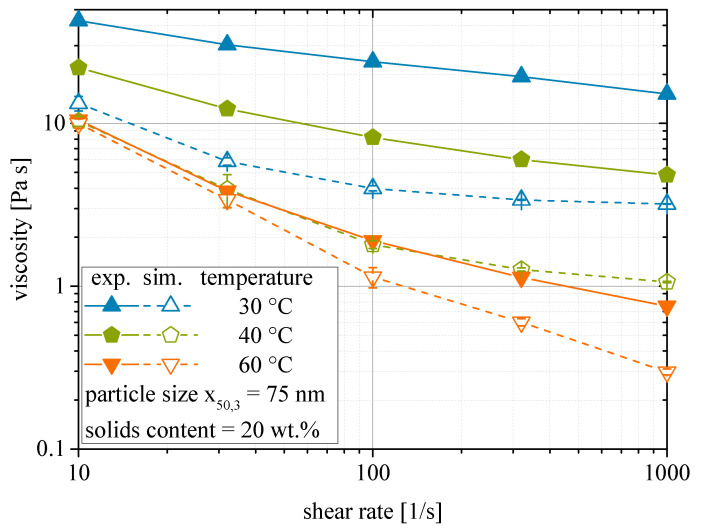
Shear rate dependency of the rheological behavior of nanoparticulate suspensions. Comparison of experimental and simulated viscosities between 30 and 60 ${}^{{}^{\circ}}$C.

Figure 5 gives the results of the same shear rate variation at lower temperatures. Although experimental and simulated curves for a temperature of 60 ${}^{{}^{\circ}}$C match at least for low shear rates, it is obvious that the simulations fail to depict the rheological behavior found in experiments at lower temperatures.

The question arises, why the temperature appears to limit the applicability of the chosen simulation method and high shear rates contribute to the inaccuracies. The viscosity of the resin under investigation shows a strong temperature dependency and increases quickly as temperature decreases. Hydrodynamic forces are known to gain increasing relevance in the rheological behavior of suspensions, when the fluid viscosity, shear rate and solids content are high. The impact of hydrodynamic forces is characterized by the dimensionless Péclet number, which is calculated from the ratio of hydrodynamic and diffusive forces, as given in Equation (14).

(14) $$Pe=\frac{\dot{\gamma }\cdot r^{2}}{2D}=\frac{3\pi \cdot \eta_{Fl}\cdot r^{3}\cdot \dot{\gamma }}{k_{B}\cdot T}$$

Brownian movement allows a system of particles to relax to an energetically favorable condition, in which the surface interaction potentials between the particles are minimized. Hydrodynamic forces disturb this equilibrium state, as they enforce relative movement between the particles. Therefore, the Péclet number describes to what extent hydrodynamic forces change the state from equilibrium conditions.

**Figure 6:**
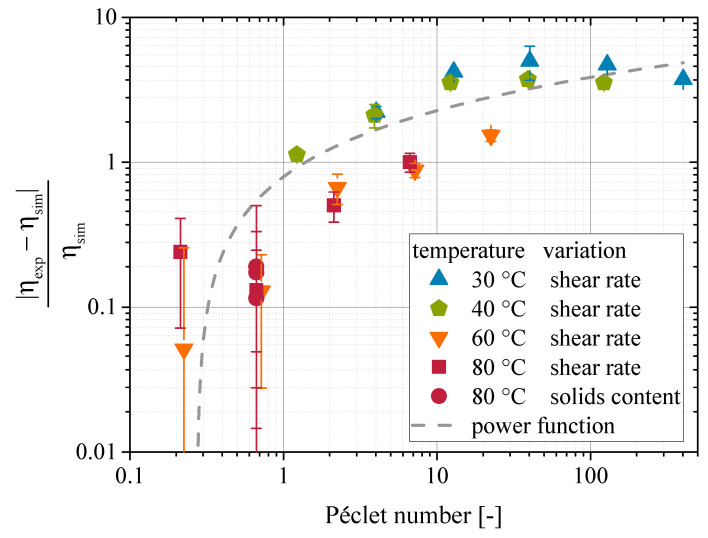
Deviations between experimental and simulated results with respect to the Péclet number.

Figure 6 displays the deviations in viscosity values between simulation and experiments with respect to the Péclet number. A normalized difference between experimental and simulated result is taken as a measure of deviation as given in Equation (15).

(15) $$\Delta \eta_{norm}=\frac{|\eta_{exp}-\eta_{sim}|}{\eta_{sim}}$$

A clear trend of increasing deviation with increasing Péclet number is visible from Figure 6. The trend can be expressed by a power function. The fact that the deviations can be described using the Péclet number suggests that hydrodynamic effects, which were not considered in the simulation, are the cause of the deviations. As mentioned earlier, the rotatory movement of the DEM-particles is not coupled with the velocity field of the fluid in the CFD. As a result, the particles experience no torque from the fluid. Also, the velocity field is not disturbed by the relative rotatory movement between the particle surface and the surrounding fluid. Lower stresses on the particles result from these effects and hence, the simulated viscosity is always lower than the experimentally observed one. Besides the lack of rotatory coupling, deviations may also arise from not regarding effects like absorbed layers of fluid molecules on the surface of the particles. This effect is commonly attributed to affect the rheological behavior of polymer suspensions, as it effects the flow behavior of fluid and particles in larger distances to the particle surface [45,46,47,48]. Such behavior has even been included in an analytical models [4]. In numerical simulations, this effect could be accounted for by an altered viscosity of the fluid close to the surface of the particles, which would require considerable extensions of the CFD-DEM code. Another approach would be to introduce another contact model in the DEM, which exhibits a normal and tangential force between the particles to cover the effect. It would work similar to the lubrication forces already used for the simulation of highly loaded micron-sized suspensions (see Chapter 2). The most challenging part would be to parametrize such contact models, as the effect is influenced by multiple parameters [4], but the precise behavior of molecules close to the surface can only be assessed by atomistic MD simulations (see for example Behbahani et al. [49]).

The identified relationship between the deviations and the Péclet number cannot only be used to explain the observed deviations, but also allows a correction of the simulated values as well. The approximated power function, which is displayed as a dashed line in Figure 6, is given by Equation (16). The values for the parameters *c*, *m*, and *e* are −21.22, 22.02 and 0.28, respectively.

(16) $$\frac{|\eta_{exp}-\eta_{sim}|}{\eta_{sim}}=c+m\cdot Pe^{e}$$

(17) $$\eta_{exp}=\eta_{corr}=\eta_{sim}+\left(c+m\cdot Pe^{e}\right)\cdot \eta_{sim}$$

**Figure 7:**
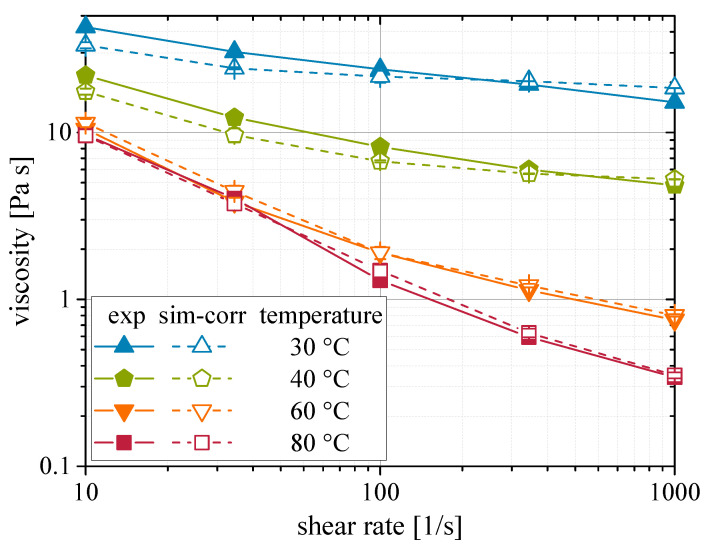
Shear rate dependency of the rheological behavior of nanoparticulate suspensions. Comparison of experimental and corrected simulated viscosities at varied temperatures.

From it, Equation (17) can be derived by solving for $\eta_{exp}$ and considering it the targeted corrected viscosity $\eta_{corr}$. Figure 7 gives the comparison of experimental and corrected viscosity values. A good fit between the respective curves is visible. It can be concluded that by calibrating a correction function with respect to the Péclet number, experimental viscosities can be matched with CFD-DEM simulations, despite the lack of rotatory velocity coupling. Nevertheless, future studies should apply solvers such as the one used by Aycock [50], which allows the coupling of rotational velocities, since shear flow imposes rotational velocities on the particles.

### 4.2. Derivation of Working Principles

The second objective of this study is to identify relationships between the acting forces in the system to identify working principles causing the observable rheological behavior. Identifying mechanistic relationships is crucial for robust viscosity models and so far, such relationships have not been established for nanoparticulate suspensions [7].

Based on the stresslet method, contact-, drag- and van der Waals forces contribute to the stress state in the suspension. The effect of contact forces is essentially zero. Because of the low solid contents, the particle insertion at 0.4 nm distance and the short-simulated time spans, particles do not have time to travel long enough to touch. Even if particles were given the time, the inertia of the small particles would not suffice to squeeze the highly viscous fluid out of the gap between the particles [51]. Consequently, only drag forces and surface interactions contribute to the rheological behavior. Elucidating their relationship helps identifying the cause of the viscosity increase due to surface forces.

**Figure 8:**
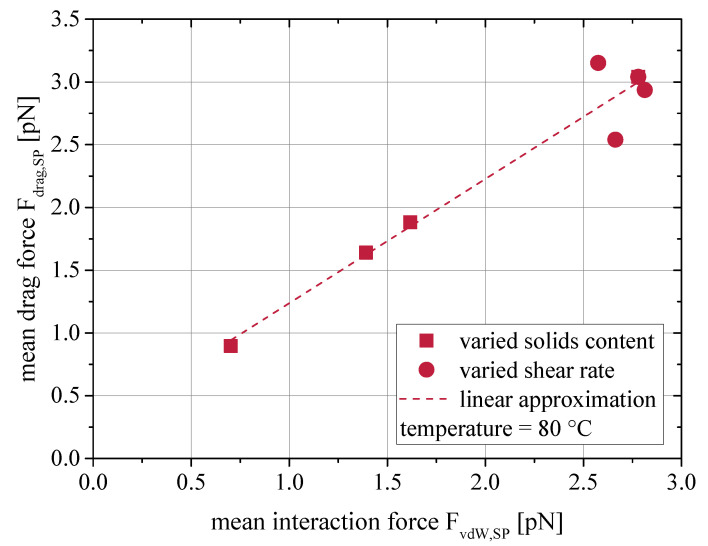
Relationship between the mean van der Waals force on a particle and the resulting mean single particle drag force in CFD-DEM simulations.

**Figure A1:**
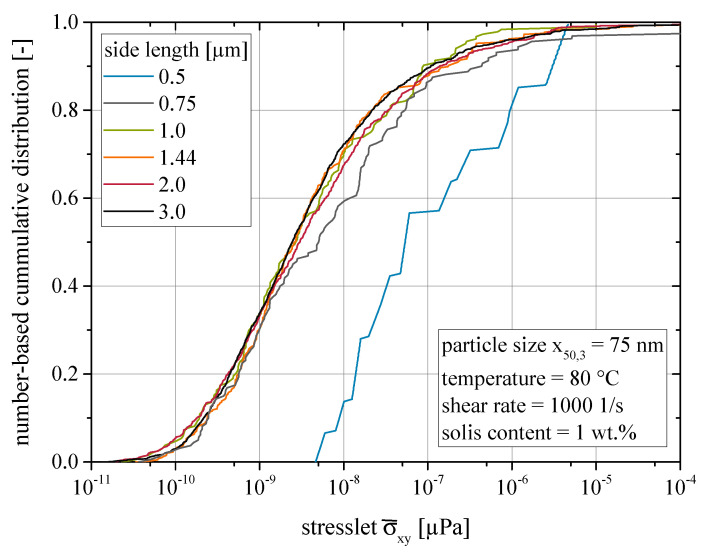
Results of the convergence study on the number-based stresslet distribution at 1 wt.%.

During the simulation, a network of surface forces is established between the particles and drag forces act on each particle. The magnitude of these forces depends on the particle size, the proximity to the next particle and the relative velocity between particle and fluid. The values of the forces span multiple orders of magnitude, much like the stresslet distribution depicted in Figure A1 in the appendix. In Figure 8 the mean per-particle drag force $F_{drag,SP}$ is plotted against the mean per-particle van der Waals force $F_{vdW,SP}$ as observed in the CFD-DEM simulations. The data of simulations at 80 ${}^{{}^{\circ}}$C is used, as it shows the best fit to experimental data. A clear linear relationship between the two forces is visible. Beyond this, the graph indicates a direct proportionality, as drag and van der Waals force are almost equal. Only the data points, which are originating from the shear rate variation, show a slight scattering, which will be assessed later. This allows the formulation of a hypothesis on the origin of such drag forces and hence the viscosity increase.

If two adjacent particles are considered, which are in different layers of a laminar flow travelling at different speeds, an interaction force occurs between them. In the case of van der Waals interactions this force is attractive. The faster flowing particle thus accelerates the neighboring particle, while the particle itself is decelerated. This creates a velocity difference between the particles and the respective surrounding laminars of the fluid. This velocity difference generates the observed drag forces and causes the increased energy dissipation during the flow and therefore, a higher viscosity of the suspension. It is plausible that the flow resistance force is as strong as the surface interaction force, which caused the flow resistance force.

**Figure 9:**
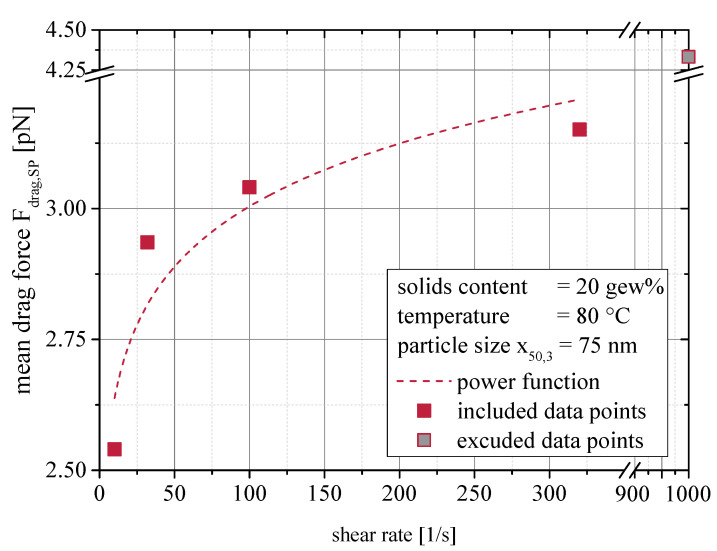
Relationship between the system shear rate and the resulting mean single particle drag force in CFD-DEM simulations.

In Figure 8 some scattering was found for the data points from the shear rate variation at 80 ${}^{{}^{\circ}}$C. This calls for a closer look onto the drag forces dependency on the shear rate, as done in Figure 9. With the exemption of the data point at 1000 1/s, a reasonable fit with a power function can be established. The approximated function is given in Equation (18). At low and moderate shear rates, the drag force grows under proportion with the shear rates. At higher shear rates, the rise of hydrodynamic forces may limit this relationship’s applicability.

(18) $$F_{drag}=2.316\cdot {\dot{\gamma }}^{0.056}$$

This proves that even when surface forces dominate the rheological behavior of nanoparticulate suspensions, hydrodynamic forces contribute significantly as well. The strong effect on the viscosity at comparatively low solids contents must be attributed to the imposed drag forces.

Based on these findings, a more mechanistic model for the viscosity of nanoparticulate suspensions is proposed, which will be developed further and investigated in future research work. A first approach is given by Equation (19), which can serve as a framework for such a model.

(19) $$\eta_{susp}=\eta_{vol}+\eta_{interaction}$$

The first term in Equation (19) accounts for the modest viscosity increase, which is caused by the presence of particles, as the simulation without surface interaction depicted. This term will most likely be expressed best by existing models like Einstein [42,43], Krieger-Dougherty [52] or Maron-Pierce [53], which already include the effect of the neat fluid viscosity, which is why an additional term including $\eta_{vol}$ will not be necessary in contrast to the way the simulations were analyzed in this paper (Equation (8)). The second term $\eta_{interaction}$ accounts for the viscosity increase due to the particle interactions as identified in this study. Thus, it will depend on the drag forces between fluid and particles, which are related to the total interaction force between the particles as well as the shear rate, which the findings in Figure 8 and Figure 9 suggest.

## 5. Conclusions

Coupled CFD-DEM simulations using the immersed boundary method proved capable of depicting the rheological behavior of nanoparticulate suspensions at high temperatures. They correctly depicted the dependency of the suspension viscosity in a broad range of solids contents and shear rates. The viscosity was calculated based on the particle-particle and fluid-particle interactions. It was shown that surface forces indeed dominate the rheological behavior of nanoparticulate suspensions, as simulations without surface forces showed almost Einstein-like behavior.

Limits arise due to disregarding particle-fluid interaction caused by particle rotation. The resulting deviations between simulation and experiment could be corrected based on a correction term, which depends on the Péclet number. In future, CFD-DEM coupling solvers, which provide rotatory coupling, such as the one used by Aycock [50] should be used to avoid a correction.

Detailed examination on the relationship between the drag force and influencing parameters like van der Waals interaction and the shear rate helped identifying working principles of the rheological behavior of nanoparticulate suspensions.

A mechanism by which surface interaction forces influence the viscosity of the suspension was proposed, based on the finding that surface forces and resulting drag forces on each particle do always match in magnitude. Attractive forces between neighboring particles alter the velocity of both particles and cause the rise of drag forces, which increases the stress state within the suspension.

This model representation of the fundamental working principle as well as the identified mathematical relationships between the active forces, allowed developing an equation, which can be used as a framework for a viscosity model for nanoparticulate suspensions. It holds the perspective of being refined to a mechanistic model for the viscosity of these materials in future. 

## Figures and Tables

**Table 1:** Parameters used for the calculation of the Hamaker constant.

Parameter	Unit	Value for Boehmite	Origin	Value for Epoxy	Origin
Main UV-absorption frequency	[1/s]	$1.43\times 10^{15}$	Alemi et al. [39]	$1.58\times 10^{15}$	measured
Refractive Index	[-]	1.65	Gu et al. [40]	1.36	measured
Dielectric Constant	[-]	2.72	calculated	1.85	calculated

## Abbreviations

The following Latin abbreviations and symbols are used in this manuscript:

*A*	surface
*a*	distance between particle centers
*b*	distance between particle surfaces
*c*	fit parameter in Equation (16)
CFD	Computational Fluid Dynamics
corr	corrected
*D*	diffusion constant
DEM	Discrete Element Method
DPD	Dissipative Particle Dynamics
*e*	fit parameter in Equation (16)
exp	experimental
*F*	force
fl	fluid
*H*	Hamaker constant
*h*	Planck constant
$k_{B}$	Boltzmann constant
*m*	fit parameter in Equation (16)
$\bar{m}$	momentum flux tensor
MD	Molecular Dynamics
*n*	refractive index
norm	normalized
Pe	Péclet number
*r*	radius
sim	simulated
SP	single particle
*T*	temperature
*V*	volume
vdW	van der Waals
*W*	potential
$x_{50,3}$	median particle size of a volume-weighted (3) particle size distribution

The following Greek abbreviations and symbols are used in this manuscript:

$\dot{\gamma }$	shear rate
ϵ	dielectric constant
η	viscosity
$\bar{\sigma }$	stress tensor
σ	stresslet
$\nu_{e}$	main UV-absorption frequency
τ	shear stress

## Appendix A. Convergence Study on Required Size of the Simulation Domain

A convergence study on the required size of the representative volume element was conducted in order to assure size-independent results of low scattering. The key to size-independent results is an appropriate representation of the particle size distribution and hence the interactions between the particles. Small simulation domains may not allow enough particles to be inserted, which leads to distorted particle size distributions and insufficiently resolved interactions. This is especially critical at low solids contents, as the number of particles per system volume is the lowest. Consequently, the lowest covered solids content of 1 wt.% was considered in the convergence study. The key value taken for evaluation of the viscosity are the stresslets as defined in Equation (10). For this reason, a convergence of the stresslet distribution of the particles within the simulation domain was taken as the criterion for a sufficient size of the representative volume element.

Figure A1 gives the stresslet distributions for varied system sizes. It is evident that at side lengths between 0.5 and 1.0μm the position of the curves changes significantly. Furthermore, a smoothing of the curves is visible. Above 1.44μm, which corresponds to three times the volume of a system with a side length of 1.0μm, no significant changes to the stresslet distribution can be observed. Hence, 1.44μm was chosen as the system side length in this study. The results depicted in Figure 3, Figure 4 and Figure 5 prove that the scattering of the results is also in a very good range.
